# Comparative deletion mapping at 1p31.3-p32.2 implies *NFIA* responsible for intellectual disability coupled with macrocephaly and the presence of several other genes for syndromic intellectual disability

**DOI:** 10.1186/s13039-016-0234-z

**Published:** 2016-03-17

**Authors:** Jonathan D. J. Labonne, Yiping Shen, Il-Keun Kong, Michael P. Diamond, Lawrence C. Layman, Hyung-Goo Kim

**Affiliations:** Department of Obstetrics & Gynecology, Augusta University, 1120 15th Street, Augusta, GA 30912 USA; Department of Neuroscience and Regenerative Medicine, Medical College of Georgia, Augusta University, 1120 15th Street, Augusta, GA 30912 USA; Department of Neurology, Massachusetts General Hospital, Harvard Medical School, Boston, MA 02114 USA; Department of Animal Science, Division of Applied Life Science (BK21plus), Institute of Agriculture and Life Science, Gyeongsang National University, Jinju, Gyeongsangnam-do Korea; Neuroscience Program, Medical College of Georgia, Augusta University, Augusta, GA 30912 USA

**Keywords:** 1p microdeletion, Intellectual disability, Delayed psychomotor development, Craniofacial anomalies, *DAB1*, *HOOK1*, *NFIA*, *DOCK7*, *DNAJC6*, *PDE4B*

## Abstract

**Background:**

While chromosome 1 is the largest chromosome in the human genome, less than two dozen cases of interstitial microdeletions in the short arm have been documented. More than half of the 1p microdeletion cases were reported in the pre-microarray era and as a result, the proximal and distal boundaries containing the exact number of genes involved in the microdeletions have not been clearly defined.

**Results:**

We revisited a previous case of a 10-year old female patient with a 1p32.1p32.3 microdeletion displaying syndromic intellectual disability. We performed microarray analysis as well as qPCR to define the proximal and distal deletion breakpoints and revised the karyotype from 1p32.1p32.3 to 1p31.3p32.2. The deleted chromosomal region contains at least 35 genes including *NFIA*. Comparative deletion mapping shows that this region can be dissected into five chromosomal segments containing at least six candidate genes (*DAB1*, *HOOK1*, *NFIA*, *DOCK7*, *DNAJC6*, and *PDE4B*) most likely responsible for syndromic intellectual disability, which was corroborated by their reduced transcript levels in RT-qPCR. Importantly, one patient with an intragenic microdeletion within *NFIA* and an additional patient with a balanced translocation disrupting *NFIA* display intellectual disability coupled with macrocephaly.

**Conclusion:**

We propose *NFIA* is responsible for intellectual disability coupled with macrocephaly, and microdeletions at 1p31.3p32.2 constitute a contiguous gene syndrome with several genes contributing to syndromic intellectual disability.

## Background

Interstitial microdeletions of the short arm of chromosome 1 are rare and to date at least 19 cases have been reported [1–17] (Table 1). It is thought that many genes, which are critical for early development, reside within the short arm of chromosome 1 [4]. These heterozygous deletions, in some cases, might have resulted in the eventual death of the affected individuals [3, 7, 9, 11]. Patients with 1p interstitial microdeletions display a number of clinical features including intellectual disability, craniofacial anomalies, seizures, tooth abnormalities, urinary tract anomalies, and skeletal, cardiac as well as limb defects [3, 4, 7, 12, 16, 17].

**Table 1:** Patients with 1p interstitial microdeletions

Patient	Karyotype	Source	Phenotype	Methods and coordinates
1	del(1)(p21p32)	[1]^a^1979	Severe psychomotor retardation, craniofacial and skeletal anomalies, short stature, overweight.	Karyotyping
2	del(1)(p22.1p31.2)	[2]^a^1987	Psychomotor retardation, craniofacial and limb anomalies.	Karyotyping
3	del(1)(p22.1p31.2)	[3]^a^1991 Pt 1	Developmental delay, intellectual disability, craniofacial anomalies.	Karyotyping
4	del(1)(p22.3p31.3)	[3]^a^1991 Pt 2	Developmental delay, seizures craniofacial and limb anomalies.	Karyotyping
5	del(1)(p32.3p34.1)	[4]^a^1991	Mental and motor developmental delay, craniofacial and limb anomalies, hypotonia.	Karyotyping
6	del(1)(p36.1p36.2)	[5]^a^1993	Developmental delay, craniofacial anomalies, neuroblastoma.	Karyotyping
7	del(1)(p32.1p32.3)	[6]^a^1995	Developmental delay, craniofacial and limb anomalies.	Karyotyping
8	del(1)(p21p22.3)	[7]^a^1997	Craniofacial and limb anomalies, congenital heart disease, rib abnormalities.	Karyotyping
9	del(1)(p34.1p34.3)	[8]^a^1999 Pt 1	Severe learning disability, attention deficit disorder (ADD), craniofacial anomalies.	Karyotyping and FISH with probes specific for chromosome 1 (COATA-SOME^TM^1, p5205-DG.5)
10	del(1)(p34.1p34.3)	[8]^a^1999 Pt 2	Attention deficit hyperactivity disorder (ADHD), craniofacial anomalies, disturbed behaviors.	Karyotyping and FISH with probes specific for chromosome 1 (COATA SOME^TM^1, p5205-DG.5)
11	del(1)(p32.1p32.3)	[9]^a^ 2002 Pt 1	Global developmental delay, craniofacial anomalies, absence of corpus callosum, type I Chiari malformation, tethered cord.	Karyotyping and FISH with probes specific for whole chromosome painting
12	del(1)(p32.1p32.3)	[9]^a^2002 Pt 2	Intraventricular hemorrhage, seizures, thin corpus callosum, limb anomalies	Karyotyping and FISH with probes specific for whole chromosome painting
13	del(1)(p32.1p32.3)	[10]^a^2003	Delayed psychomotor development, craniofacial anomalies	Karyotyping and FISH using whole chromosome 1 painting probe (wcp1)
14	del(1)(p36)	[11]^a^2004	Craniofacial anomalies, moderate intellectual disability, seizures.	FISH with TelVysion 1p (Vysis), P5124 (Oncor), a YAC 273d11 (CEPH)
15	del(1)(p31.3p32.2)	[12] 2010	Craniofacial anomalies, hypoplasia of corpus callosum, ventriculomegaly, hypotonia	105K Oligoarray CGH & BAC array CGH, 4.93 Mb deletion, 1p32.2p31.3 (chr1:58,193,565-63,125,273)X1 *dn*, hg19
16	del(1)(p36.3)	[13]^a^ 2010	Prader-Willi like features.	MLPA, FISH with BAC probes, real-time q-PCR
17	del(1)(p31.1p32.2)	[14] 2014	Craniofacial anomalies, partially hypoplastic corpus callosum, mild ventriculomegaly, intraparenchymal hemorrhages, cerebral palsy.	SNP- microarray (SNP-CMA), 22.9 Mb deletion (chr1:55,113,975-77,992,492)X1, hg19
18	del(1)(p36.3)	[16] 2008	Developmental delay, facial dysmorphisms, neuroblastoma	MLPA, 244 K oligo microarray, a deletion of 1.59 Mb at 1p36.3 and a duplication of 3.26 Mb at 1p36.3 (1,741,058-5,004,693)X3, hg18, FISH with Vysis 1p36 and BACs
19^b^	del(1)(p31.3p32.1)	[17] 2007	Abnormal corpus callosum, Ventriculomegaly, developmental tethered spinal cord, Chiari I malformation	array Comparative genomic hybridization (aCGH)
20	del(1)(p32.1p32.3)	[22] 2015	Microcephaly, urogenital anomalies, hearing loss, choanal atresia.	SNP microarray, 6.4 Mb deletion (chr1:54,668,618-61,113,264)X1, hg19, FISH with BACs
DCP274057	partial C-terminal duplication of *NFIA*	DECIPHER	90 kb duplication at 1p31.3, global developmental delay	
DCP260253	intragenic deletion of *NFIA*	DECIPHER	117 kb deletion at 1p31.3, phenotype not available	
DCP285169	intragenic duplication of *NFIA*	DECIPHER	419 kb duplication paternally inherited at 1p31.3, additional 2.07 Mb maternal deletion at 15q13, delayed fine motor development, expressive language delay, impaired social interactions, receptive language delay	
DCP288170	intragenic *NFIA* deletion	DECIPHER	229 kb deletion at 1p31.3, intellectual disability, macrocephaly	
DCP300407	del(1)p32.1	DECIPHER	281 kb deletion at 1p32.1, cognitive impairment	
DCP264827	del(1)p31.3	DECIPHER	5.43 Mb deletion at 1p31.3, abnormally folded helix, ADHD, constipation, flat forehead, global developmental delay, hypothyroidism, malar flattening	
DCP276512	del(1)p31.3p32.1	DECIPHER	8.83 Mb deletion at 1p31.3-1p32.1, delayed speech and language	

^a^ Microdeletions without genomic coordinates reported in the pre-microarray era. Pt denotes patient

^b^Pt 19 has additionally a chromosomal translocation 46,XY,t(1;3)(p22;q21)dn

Revisiting CNV (copy number variation) cases reported during the pre-microarray era is uncommon, because the patients are deceased or lost to follow-up. Re-evaluation of earlier cases using microarray analysis, however, provides more accurate information on the candidate genes contained within the CNV regions. Furthermore, it also allows monitoring of any changes in the patient’s clinical features over time [18]. More than a decade ago, an apparent interstitial microdeletion at 1p32.1p32.3 was reported in a 10-year old girl with delayed psychomotor development and facial dysmorphism. This *de novo* structural chromosomal rearrangement was detected by karyotype analysis alone followed by fluorescent *in situ* hybrization (FISH) without defining the exact size and location of the microdeletion [10]. Here we revisited this proband to revise the karyotype and to define the size of this microdeletion by molecular analysis. From molecular dissection of our case with six published cases of microdeletions, a balanced translocation, and seven unpublished DECIPHER cases [23], we propose that there are at least six chromosomal segments within this region, and each segment harbors at least one candidate gene for intellectual disability. We also determined the transcript levels of six candidate genes (*DAB1*, *HOOK1*, *NFIA, DOCK7*, *DNAJC6* and *PDE4B*) for syndromic intellectual disability.

## Methods

### Case presentation

**Fig. 1:**
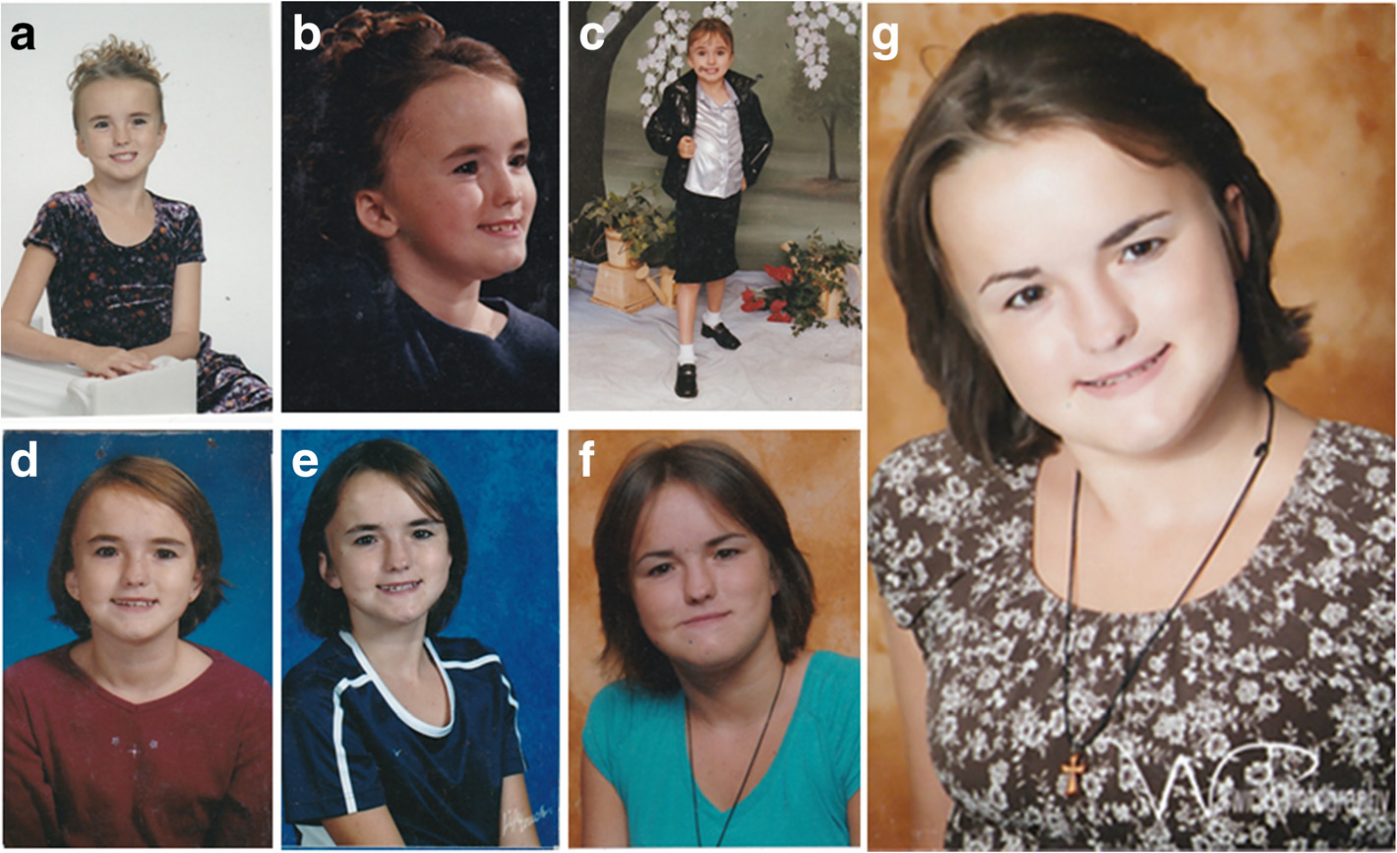
Phenotypic features of DGDP005. **a** Facial and head appearance including macrocephaly and upper body picture at 4 years (**b**) Lateral facial feature of 4 years of age showing frontal bossing (**c**) Full body picture as a 7 years old (**d**) upper body and head appearance presenting with macrocephaly at 12 (**e**) 13 (**f**) 16 and (**g**) 18 years of age

The proband (DGDP005) is a 23-year-old female with a history of developmental delay and intellectual disability. In infancy, developmental milestones were delayed and she exhibited impaired motor skills (Table 2). The patient displayed craniofacial anomalies including macrocephaly (Fig. 1a, b, d-g), frontal bossing (Fig. 1b) and low-set ears (Fig. 1d). She was diagnosed with attention deficit hyperactivity disorder (ADHD), obsessive compulsive disorder (OCD), ocular hypertension (OHT), and developmental encephalopathy with cognitive impairment (Table 2).

**Table 2:** Timescale of DGDP005’s medical evaluations as well as her clinical features

Age	Evaluation	Clinical features
<5 years	Pediatrician	- Crawling, sitting, walking and attaining language milestones delayed
		- Craniofacial anomalies including macrocephaly
		- Problematic motor skills.
~10 years	Developmental pediatrician	- Special education implemented in curriculum
		- Difficulty falling asleep and treatment with melatonin
		- ADHD
		- Hypertonia.
		- Possible sensory motor difficulties.
14 years	Standford Binet Intelligence Scales-Fifth edition (SB5)	- Difficulty staying on the task and needed to be redirected from time to time
		- Overall thinking and reasoning at ~13 percentile.
		- Borderline nonverbal reasoning abilities
		- Verbal reasoning at ~12 percentile
		- Verbal and nonverbal problem solving at ~ 58 percentile
		- Ability to gather information at ~ 18 percentile
		- Numerical problem solving at ~7 percentile.
		- Borderline visual display abilities
		- Ability to maintain attention at ~13 percentile.
		- Full scale quotient of 83 (95 % confidence interval 79–87) suggesting intellectual disability.
16 years	Individualized education program review team	- OHT
		-ADHD
		- Language disability in written language expression
		- Disability in math calculation and reasoning
		- Difficulties in fine graph motor functions with soft neurological signs.
		- Continues to be a student with disability
18 years	Child neurology services	- Developmental encephalopathy
		- Cognitive impairment
		- OCD as well as ADHD
		-Treatment with fluoxetine (aka Prozac10 mg) and Adderall (15 mg)
		- More pronounced facial dysmorphisms including prominent forehead, low-set ears, narrow nose thin lips
22 years	Admitted to emergency	- Diagnosed with intraventricular hemorrhage with layering in the posterior fossa.
		- Right anterior communicating artery aneurysm
		- Developmental delay, OCD and ADHD

At age 10, her grades were Bs and Cs, but school challenges warranted special education. She also had difficulty falling asleep and she took nightly melatonin. She was diagnosed with ADHD, hypertonia, and possible sensory integration difficulties (further details on her phenotype up to age10 can be found in Zinner and Batanian (2003) [10]).

At 14 years of age, she took the Stanford Binet Intelligence Scales-Fifth edition (SB5). She had difficulty staying on a task requiring redirection. Her general cognitive ability was estimated to be in the low average range with a full scale intelligence quotient (FSIQ) score of 83 (95 % confidence interval 79–87), suggesting intellectual disability. She received a score of 79 in the nonverbal reasoning abilities, which is in the borderline range, and higher than only 8 % of her peers. In verbal reasoning abilities, she scored 88, which is in the low average, and just above ~12 % of her peers. Her ability to solve verbal and nonverbal problems using inductive or deductive reasoning was average. Her overall thinking and reasoning was worse than ~87 % of children of her age. She scored in the low average range for general information gathering, attention span, and concentration. Her ability to see patterns, relationships, and spatial orientations among diverse pieces of visual display, as well as her ability with numbers and numerical problem solving with word problems or with picture relationships were borderline.

At age 16, she was found to display OHT and ADHD, language disability in written language expression as well as in math calculation and math reasoning. She passed the vision test scoring 20/10 and 20/20 for the right and left eye respectively. Her hearing was adequate for the classroom functioning. She showed significant difficulties in fine motor functions with soft neurological signs. Overall, the assessment showed that she continued to be a student with intellectual disability and ADHD.

When she was 18, the patient was re-evaluated by the child neurology services. She was diagnosed with developmental encephalopathy with cognitive impairment, OCD and ADHD. She was treated with fluoxetine and Adderall for OCD and ADHD, respectively. Some of her facial dysmorphisms became more evident at age 18, such as her prominent forehead, narrow nose, and thin lips (Fig. 1). She still displayed low-set ears (Fig. 1d). At the age of 22, she was admitted to the hospital and diagnosed with subarachnoid as well as intraventricular hemorrhage with layering in the posterior fossa. She was also diagnosed with a right anterior communicating artery aneurysm and continued to show developmental delay, OCD and ADHD.

### Cell culture

Lymphocytes were isolated from the patient’s blood samples by density gradient centrifugation as we have done previously [19]. Lymphoblastoid cell line (LCL) was generated from the patient’s blood in order to determine the transcript levels of genes of interests.

### Genomic DNA extraction and microarray

Genomic DNA was extracted from the patient’s blood samples using a standard phenol-chloroform protocol with minor modifications. The human genome 244 K array (Agilent technologies, G4411B) was used to detect copy number variation as described previously in Miller et al. (2012) [20].

### Real-Time genomic qPCR and RT-qPCR

Primers targeting regions encompassing the proximal and distal deletion breakpoints were designed for qPCR. Primer pairs were first evaluated by determining the primer efficiency using a serial dilution of genomic DNA as template.

Primers were also designed against exonic regions of genes of interests for RT-qPCR. Total RNA was isolated from cell lines established from patient DGDP005 using the RNeasy Plus Mini kit (Qiagen) following the manufacturer’s instructions. cDNA was synthesized from 1 μg of total RNA using the RevertAid First cDNA Synthesis Kit (Thermo Scientific) according to the manufacturer’s protocol. All Real-Time qPCR reactions were carried out using 2 μl of sample, 10 ml of Fast Essential DNA Green Master (Roche) and 2.5 μM primers in a total reaction volume of 20 μl.

## Results

### Microarray

**Fig. 2:**
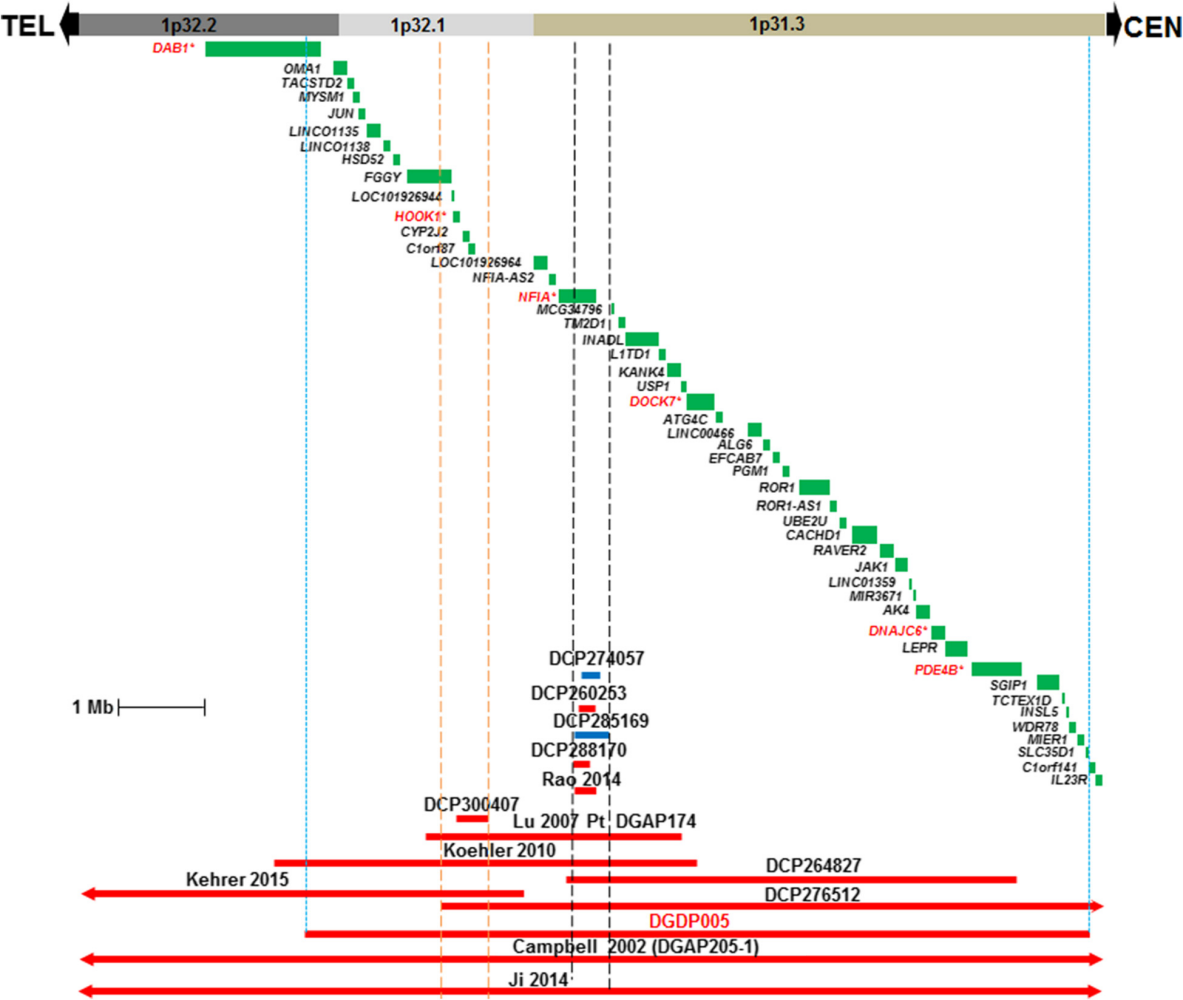
Comparative deletion mapping of patients with CNVs at 1p31.3p32.2. The genes located within this interval are displayed. Microdeletions are represented by solid red bars while microduplications are displayed in blue. Patients with CNVs from the DECIPHER database (Firth et al., 2009) are denoted by ‘DCP’ followed by the reference number. The sizes of the deleted regions in Rao 2014, Lu 2007 (DGAP174), Koehler 2010, Kehrer 2015, Campbell 2002 (DGAP205-1), Ji 2014 and seven DECIPHER cases relative to our patient (DGDP005) are displayed. Four CNVs (DCPP274057, DCP260253, DCP288170, Rao 2014) have only *NFIA* disrupted implying that it is causative gene in all four patients. Patient DCP285169 with a 419 kb duplication including the second half of *NFIA* presents with language delay, impaired social interactions, and delayed motor development. But he also has an additional 2.07 Mb microdeletion at 15q13. The microdeletion in patient DCP300407 does not include *NFIA*, yet the patient displays cognitive impairment, suggesting the likely contribution of *HOOK1*. Two microdeletions (Ji 2014 and Campbell 2002) extend beyond the 1p32.2 and 1p31.3 interval. Vertical lines in blue represent the proximal and distal boundaries of the microdeletion in DGDP005. Two orange lines are refined candidate gene region overlapping eight microdeletions including DCP300407. Two vertical black lines depict narrowed candidate region among 5 small CNVs [4 DCP cases and patient of Rao et al. (2014)]. Asterisk denotes candidate genes in this region

Microarray performed on genomic DNA derived from patient DGDP005 revealed a 9.45 Mb microdeletion at 1p31.3p32.2 (chr1: 57,633,718- 67,087,056, GRCh38/hg38). The deleted chromosomal region contains at least 35 genes, including *NFIA* (Fig. 2).

### Comparative deletion mapping

We compared all clinical features displayed by DGDP005 with five previously reported microdeletion cases namely, Campbell et al. (2002) [9], Koehler et al. (2010) [12], Ji et al. (2014) [14], Lu et al. (2007) [17], Rao et al. (2014) [21], Kehrer et al. (2015) [22] at 1p31.1p32.2 overlapping our microdeletion at 1p31.3p32.2, as well as one translocation case involving the *NFIA* gene in Lu et al. (2007) [17] (Table 3). We also compared the microdeletion in DGDP005 to seven cases from the DECIPHER database [24] (Fig. 2, Table 1). The refined candidate gene region represented by DCP300407 and proximal breakpoint of DCP276512 was depicted in two orange lines (Fig. 2), while two vertical black lines depict narrowed candidate region among 5 small CNVs including 4 DCP cases and a microdeletion of Rao et al. (2014) [21]. We examined the functions of all genes involved in the microdeletion by reviewing the literature to highlight candidate genes that could contribute to phenotypes observed in DGDP005 (Table 4). Patient DGAP174 of Lu et al. (2007) was not included in determining genotype-phenotype relationships (Table 3), because of an accompanying chromosomal translocation t(1;3) (p31.1; q25.1) [17].

**Table 3:** Clinical features of DGDP005 along with five additional microdeletion cases and a balanced translocation t(1;20)(p31.3;q13.31)*dn* disrupting the *NFIA* gene

Clinical features	DGDP005 del(1)p31.3 p32.2	Koehler et al. 2010 del(1)p31.3 p32.2	Campbell et al. 2002 DGAP205-1 & DGAP205-1S del(1)p31.3 p32.3	Ji et al.2014 del(1)p31.1 p32.2	Rao et. al 2014 del(1) p31.3	Lu et al., 2007 DGAP174^a^del(1)(p31.3 p32.1)	Lu et al., 2007 DGAP104 Balanced translocation t(1;20) (p31.3;q13.31)*dn*
Developmental delay	+	+	+	+	+	+	+
Intellectual disability	+	NS	+	NS	NS	+	+
Macrocephaly	+	+	+	+	+	+	+
Frontal bossing	+	-	+	NS	NS	NS	NS
Developmental encephalopathy	+	-	-	-	-	-	-
OHT	+	-	-	-	-	-	-
Intraventricular hemorrhage	+	-	+	+	-	-	-
Impaired motor skills	+	NS	+	NS	NS	+	+
Attention deficit disorder	+	NS	-	NS	NS	+	+
Hypertonia	+	NS	NS	NS	NS	-	NS
OCD	+	-	+	-	NS	-	-
Seizures	-	-	+	+	-	NS	NS
Abnormal corpus callosum	NS	+	+	+	+	+	+
Ventriculomegaly	-	+	+	+	+	+	-
Tethered spinal cords	-	-	+	-	-	+	+
Chiari I malformation	-	NS	+	-	-	+	+
Urinary tract defects	-	-	+	+	+	NS	-

NS: Not Stated; Pt: Patient

^a^DGAP174 also has an additional chromosome translocation, 46,XY, t(1:3)(p31.1;q25.1)*dn*

**Table 4:** Functions of candidate genes possibly contributing to clinical phenotypes observed in patient DGDP005

Gene name	Gene symbol	Age	Association with neuro-developmental features	Remarks
Nuclear factor I/A	*NFIA*	[16] ~2 yrs	Abnormal corpus callosum, ventriculomegaly, hydrocephalus, developmental delay, tethered spinal cord, chiari I malformation, and urinary track defect	Disruption of *Nfia* in mice result in severe developmental defects including agenesis of the corpus callosum, severe communicating hydrocephalus, neurological defects, male sterility, female subfertility [24]
		[21] 8 yrs		
Dab, reelin signal transducer homolog 1	*DAB1*	[37] ~7.5 yrs	Autism	
Hook homolog 1(Drosophila)	*HOOK1*	[23] 5 yrs	Cognitive impairment (DCP300407)	Cytosolic protein possessing a conserved N-terminal domain that binds to microtubules. Interacts with CLN3, the causative gene for autosomal recessive Batten disease.
Dedicator of cytokinesis 7	*DOCK7*	[38] 5–7 yrs	Epileptic encephalopathy, dysmorphic features and intellectual disability	*DOCK7* plays a role in neural development
DnaJ (Hsp40) homolog, C, member 6	*DNAJC6*	[31] 17–44 yrs	Parkinson disease Subfamily	-
Phosphodiesterase 4B, cAMP-specific	*PDE4B*	[35] NS	Schizophrenia	-

NS; Not stated

### Real-Time qPCR

**Fig. 3:**
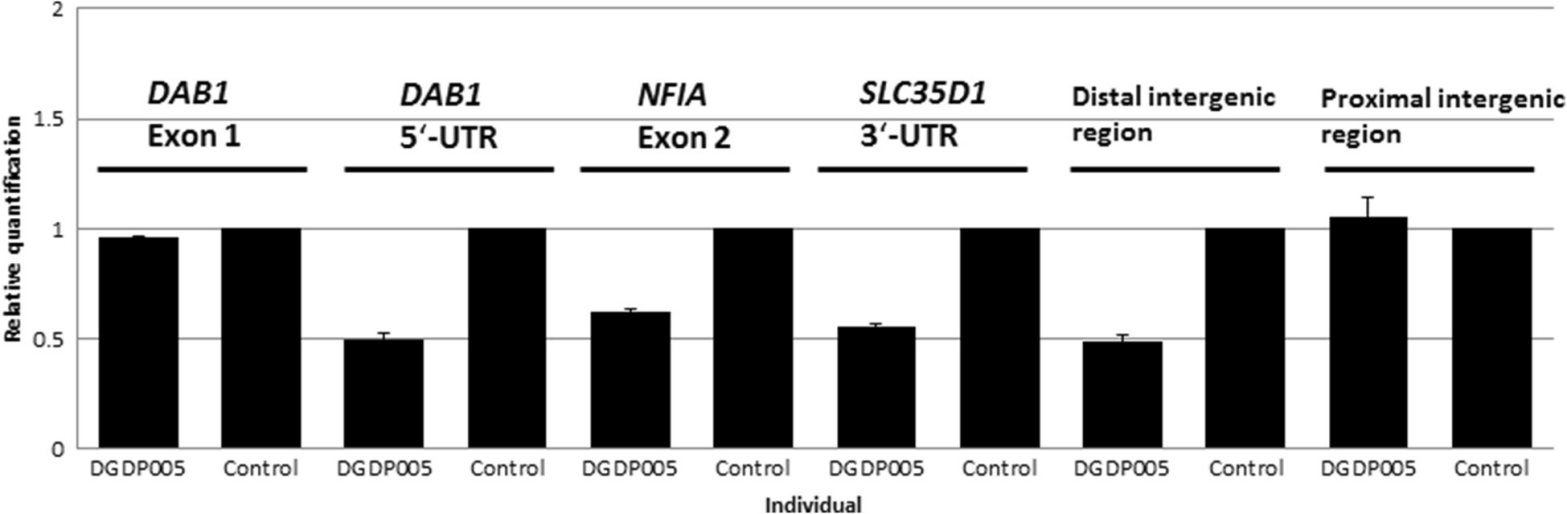
Refining the deletion breakpoints in DGDP005 by qPCR. The 5′-UTR of the *DAB1* gene is deleted while its first exon is intact indicating that the distal deletion breakpoint lies between *DAB1* exon 1 and the 5′-UTR. The *NFIA* gene and *SLC35D1* are both completely deleted in our patient. Two separate loci located in the proximal and distal intergenic region between *SLC35D1* and *C1orf141* were also assayed by qPCR. The distal intergenic region residing closer to *SLC35D1* was found to be contained in the microdeletion, while the proximal intergenic region was intact. A value close to 1 indicates that the locus is not deleted, while a value near 0.5 shows deletion on another allele

We performed qPCR to refine the flanking coordinates of the deletion breakpoints. qPCR assays using primers designed from the *DAB1* gene revealed that the distal deletion breakpoint is located between *DAB1* exon 1 and its 5′-UTR. The *SLC35D1* gene was found to be completely deleted as predicted by microarray (Figs. 2 and 3). The proximal deletion breakpoint was found to reside in the intergenic region between gene *SLC35D1* and uncharacterized gene *C1orf141* (Figs. 2 and 3).

### RT-qPCR

**Fig. 4:**
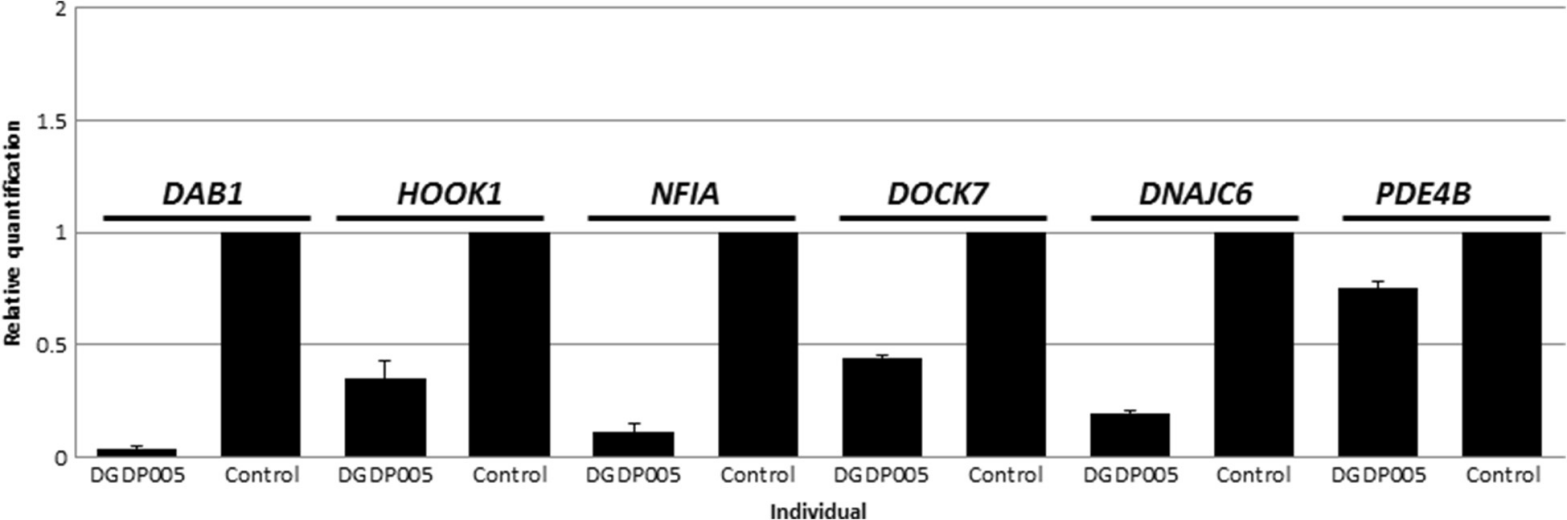
Transcript levels of six candidate genes involved in CNVs were determined by RT-qPCR. The level of transcripts of *DAB1*, *NFIA* and *DNAJC6* were markedly reduced in DGDP005 relative to the healthy white female control. Transcripts derived from *HOOK1* and *DOCK7* were reduced approximately by half, while *PDE4B* transcripts decreased moderately

Expression analysis of six genes of interest by RT-qPCR revealed that the transcript levels of *NFIA*, *DAB1* and *DNAJC6* were markedly reduced in the patient relative to a healthy white female control (Fig. 4). Transcripts derived from *HOOK1* and *DOCK7* were reduced approximately by half, while *PDE4B* transcripts decreased moderately (Fig. 4).

## Discussion

The female patient (DGDP005) examined in this study was first reported as a 10-year old girl possessing a rare 1p32.1p32.3 microdeletion [10]. Characterization of the microdeletion in the patient by microarray allowed the refinement of the microdeletion from 1p32.1p32.3 to 1p31.3p32.2. The health condition of DGDP005 has been monitored regularly since the first few months of her life. Psychomotor delay and craniofacial anomalies were apparent since infancy [10]. During the follow-up, she has continued to display developmental delay and as a result, special education has been implemented in her curriculum. Craniofacial anomalies such as macrocephaly and frontal bossing became more pronounced over time, suggesting the involvement of culprit gene(s) in continuous skull development since infancy [10]. Additional features such as OCD and intraventricular hemorrhage were also observed after the age of ten, emphasizing the importance of follow-up.

We have now determined the size of the deletion in DGDP005 by microarray and refined the locations of the flanking deletion breakpoints by qPCR (Figs. 2 and 3). The deleted region is at least 9.45 Mb encompassing more than 35 annotated genes including *NFIA* [17]. Only four smaller-sized heterozygous microdeletions (Rao 2014, Lu 2007, Koehler 2010 and Kehrer 2015) have been reported so far within this interval (Fig. 2). The patient of Koehler et al. (2010) [12] had a 1p31.3p32.2 microdeletion while another patient DGAP174 of Lu et al. (2007) had a deletion at 1p31.3p32.1 with additional translocation, 46,XY, t(1;3)(p31.1;q25.1) [17]. The patient of Rao et al. (2014) had a small microdeletion spanning exons 4–9 within *NFIA* [21]. The distal part of the microdeletion of Kehrer overlaps with the proximal part of DGDP005 and the more refined candidate region is represented by DCP300407. Two larger microdeletions of two half siblings (DGAP205-1 and DGAP205-1S) (Table 3) reported in Campbell et al. (2002) and a patient described in Ji et al. (2014) encompassing the whole chromosomal region deleted in DGDP005, have also previously been published [9, 14] (Fig. 2).

Haploinsufficiency of the *NFIA* gene is expected to cause an abnormal corpus callosum, ventriculomegaly or hydrocephalus, developmental delay, tethered spinal cord, Chiari I malformation, seizures, urinary tract defects, and craniofacial anomalies [17, 21]. Furthermore, disruption of *nfia* in mouse results in severe developmental defects including agenesis of corpus callosum, severe communicating hydrocephalus, female subfertility, and male sterility [24]. Five CNV cases (DCDP274057, DCP260253, DCP285169, DCP288170, Rao 2014) only had *NFIA* disrupted (Fig. 2). Among these five DECIPHER cases, DCP288170 with an intragenic deletion within *NFIA*, displayed intellectual disability. Although, this phenotype was not emphasized in Lu et al. (2007) [17], DGAP104 with a balanced translocation of t(1;20)(p31.3;q13.31) disrupting *NFIA* at 1p31.3 breakpoint (Table 3) also displayed intellectual disability (global IQ score of 52). Patient DCP274057 presents with global developmental delay while DCP285169 displays expressive and receptive language delay. The patient reported in Rao et al. (2014) with a deletion involving exons 4–9 of *NFIA* was only 8 years-old, and had not yet been evaluated intellectual disability [21]. The aforementioned patients clearly suggest that disruption of *NFIA* is likely to cause intellectual disability, or cognitive deficits. Indeed, the *NFIA* transcript levels were markedly reduced in DGDP005 (Fig. 4) suggesting that *NFIA* may be contributing to syndromic intellectual disability.

Notably, four microdeletion patients namely Campbell et al., 2002 [9], Koehler et al., 2010 [12], Ji et al., 2014 [14] and DGDP005 had in common macrocephaly (Table 3). Importantly, a balanced translocation patient DGAP104, in whom *NFIA* is disrupted at 1p31.3 [17] also had macrocephaly. Furthermore, the patient reported in Rao et al. (2014) [21] with a smaller microdeletion within *NFIA* also had macrocephaly (Fig. 2 and Tables 1 and 3). Collectively, these findings underscore that the haploinsufficiency of NFIA is sufficient to cause intellectual disability and macrocephaly, two of the core phenotypic features of del (1)(p31.3p32.2) microdeletion syndrome.

While comparing the clinical features of our patient with the two half-siblings [9] who have a larger microdeletion including *NFIA*, we found that DGDP005 does not manifest ventriculomegaly, tethered spinal cord, Chiari I malformation, or urinary tract defects. Similarly, the patient having a smaller microdeletion encompassing *NFIA* [12] displayed only three of the clinical features as a result of *NFIA* deletion, namely abnormal corpus callosum, ventriculomegaly and developmental delay. Incomplete penetrance may partly explain why all phenotypes expected from *NIFA* disruption are not manifested in the patients with an *NFIA* deletion (Table 3 and Fig. 2) [25]. We have not included DGAP174 [17] in the comparison because he had a *de novo* translocation t(1;3) (p31.1; q25.1)*dn* in addition to a 2.2 Mb interstitial deletion at 1p31.3-p32.1 (Table 3).

Reviewing phenotypes displayed by patients with microdeletions at 1p31.3-1p32.2 enabled identification of at least 17 clinical features seen across all patients (Table 3). Because of the putative chromosomal segments with corresponding candidate genes for cognitive impairment within this region, it is obvious that not all phenotypes observed can be attributed to *NFIA* alone. It is likely that genes including *NFIA* in this region are haploinsufficient thereby contributing to the 17 clinical features observed across all patients (Table 3) [17]. It supports the idea that the 1p31.3p32.2 microdeletion may constitute a “chromosome syndrome” postulated in Zinner and Batanian (2003) [10], which meant to be contiguous gene deletion syndrome [26]. Recently, Ji et al. (2014) [14] also postulated the possible involvement of genes other than *NFIA*. We have reviewed the functions of all genes within the 1p31.3p32.2 microdeletion in DGDP005. Molecular dissection of this region suggests the presence of at least five chromosomal segments, with six candidate genes for intellectual disability. Each segment contains *DAB1*, *HOOK1*, *NFIA*, *DOCK7*, and *DNAJC6* as well as *PDE4B*, respectively. We propose six candidate genes (*DAB1*, *HOOK1*, *DOCK7*, *DNAJC6*, *PDE4B*, and *NFIA*) for the clinical features such as intellectual disability and OCD observed in DGDP005, because each of these genes is involved directly or indirectly in neurological disorders (Table 4).

While HOOK1 interacts with CLN3, the causative gene for the autosomal recessive Batten disease characterized by visual impairment, gait anomalies, seizures, dementia and sometimes mental deterioration [27–30], *DNAJC6* has been shown to be implicated in Parkinson disease [31]. It is noteworthy that Batten disease is also characterized by progressive neurodegeneration and impaired motor skills [32] like Parkinson disease [33, 34]. Furthermore, patient DCP300407 with only *HOOK1*, *CYP2J2* and *C1orf87* deleted (Fig. 2) displays cognitive impairment [24]. Additionally, *PDE4B* is implicated in schizophrenia [35], whereas *DAB1* truncated in our patient is required for synaptic function as well as associative learning in mice [36], and has been shown to be associated with autism [37].

Recently, mutation in *DOCK7* has been found to be associated with epileptic encephalopathies, dysmorphic features and intellectual disability [38]. Interestingly, DGDP005 displays some of the phenotypes expected from *DOCK7* mutation indicating that *DOCK7* may also contribute to intellectual disability and craniofacial anomalies in our patient. As is the case for *DOCK7*, cognitive impairment is an overlapping clinical feature also produced by disruption in *NFIA*. This further supports the hypothesis that deletion at 1p31.3p32.2 constitutes a contiguous gene deletion syndrome. As expected, the transcript levels of *DOCK7* and *HOOK1* was reduced approximately by half. The level of transcripts of *DAB1*, *DNAJC6* and *PDE4B* were also reduced in our patient relative to a healthy white female control (Fig. 4). The significant reduction of transcripts of *NFIA* and two additional genes (*DAB1* and *DNAJC*6) suggests an allele-specific expression pattern (Fig. 4). This may be due to a loss of the allele actively transcribed in the microdeletion. It is unclear whether the causative effect of each candidate gene is stoichiometrically contributing to the manifestation of the phenotype or an effect of one gene is masked by the effect of other gene when more than one candidate gene is deleted in a microdeletion. If some of the candidate genes participate in overlapping molecular pathways leading to cognitive impairment, the effect of one gene may be masked by another.

## Conclusion

Revisiting patient DGDP005 has allowed us to refine the flanking proximal and distal breakpoints, enabling us to compare the clinical phenotypes of DGDP005 to reported cases and unpublished DECIPHER cases. Comparative deletion mapping showed that there are at least six candidate genes including, *NFIA*, *HOOK1*, *DOCK7*, *DAB1*, *DNAJC6*, and *PDE4B*, that could contribute to the syndromic or non-syndromic intellectual disability. Most importantly, the genomic and clinical delineation result implies *NFIA* responsible for intellectual disability coupled with macrocephaly. Smaller-sized microdeletions across this interval and rare point mutations in the genes should reveal a better understanding of the pathological role of individual gene residing within this region. Additionally, expression studies including haploinsufficiency of aforementioned candidate genes in this interval will provide more insights both on the number of candidate genes and their impact on this contiguous gene syndrome.

## Consent

This study was approved by the Institutional Review Board of Augusta University and written informed consent was obtained from the mother of DGDP005 for the publication of this report and accompanying images.

## Abbreviations

qPCR: quantitative polymerase chain reaction
DAB1: DISABLED, DROSOPHILA, HOMOLOG OF, 1
HOOK1: HOOK, DROSOPHILA, HOMOLOG OF, 1
NFIA: NUCLEAR FACTOR I/A
DOCK7: DEDICATOR OF CYTOKINESIS 7
DNAJC6: DNAJ/HSP40 HOMOLOG, SUBFAMILY C, MEMBER 6
PDE4B: PHOSPHODIESTERASE 4B, cAMP-SPECIFIC
CNV: copy number variation
FISH: fluorescent in situ hybridization
RT-qPCR: reverse-transcriptase quantitative polymerase chain reaction
ADHD: attention deficit hyperactivity disorder
ADD: attention deficit disorder
OCD: obsessive compulsive disorder
OHT: ocular hypertension
SB5: Stanford binet intelligence scales-fifth edition
FSIQ: full-scale intelligence quotient
DCP: DECIPHER
SLC35D1: SOLUTE CARRIER FAMILY 35 (UDP-GLUCURONIC ACID/UDP-N-ACETYLGALACTOSAMINE DUAL TRANSPORTER), MEMBER D1
C1orf141: Homo sapiens chromosome 1 open reading frame 141
